# Process–Structure–Property Relationships of Copper Parts Manufactured by Laser Powder Bed Fusion

**DOI:** 10.3390/ma14112945

**Published:** 2021-05-29

**Authors:** Mohamed Abdelhafiz, Kassim S. Al-Rubaie, Ali Emadi, Mohamed A. Elbestawi

**Affiliations:** 1 Additive Manufacturing Group (AMG), Department of Mechanical Engineering, McMaster University, 1280 Main Street West, Hamilton, ON L8S 4L7, Canada; elbestaw@mcmaster.ca; 2 Department of Electrical & Computer Engineering, McMaster University, 1280 Main Street West, Hamilton, ON L8S 4K1, Canada; emadi@mcmaster.ca

**Keywords:** additive manufacturing, laser powder bed fusion, pure copper, process–structure–property relationships, physical properties, chemical concentration

## Abstract

The process–structure–property relationships of copper laser powder bed fusion (L-PBF)-produced parts made of high purity copper powder (99.9 wt %) are examined in this work. A nominal laser beam diameter of 100 μm with a continuous wavelength of 1080 nm was employed. A wide range of process parameters was considered in this study, including five levels of laser power in the range of 200 to 370 W, nine levels of scanning speed from 200 to 700 mm/s, six levels of hatch spacing from 50 to 150 μm, and two layer thickness values of 30 μm and 40 μm. The influence of preheating was also investigated. A maximum relative density of 96% was obtained at a laser power of 370 W, scanning speed of 500 mm/s, and hatch spacing of 100 μm. The results illustrated the significant influence of some parameters such as laser power and hatch spacing on the part quality. In addition, surface integrity was evaluated by surface roughness measurements, where the optimum Ra was measured at 8 μm ± 0.5 μm. X-ray photoelectron spectroscopy (XPS) and energy-dispersive X-ray spectroscopy (EDX) were performed on the as-built samples to assess the impact of impurities on the L-PBF part characteristics. The highest electrical conductivity recorded for the optimum density-low contaminated coils was 81% IACS.

## 1. Introduction

Laser powder bed fusion (L-PBF), also known as selective laser melting (SLM), is one of the main metal additive manufacturing (AM) methods used for producing parts with complicated geometries. Pure copper has the best electrical conductivity (EC) amongst nonprecious metals. Accordingly, the EC scale of all metals is expressed as a percent of the international annealed copper standard (IACS); i.e., 100% IACS is equivalent to 58 × 10^6^ S/m for C10700 and C11300 copper alloy. Moreover, according to the Wiedemann–Franz relationship [1], thermal conductivity (TC) is strongly proportional to EC, making copper an excellent candidate for most electrical and thermal management applications. During the last decade, significant research efforts were aimed at AM of copper alloys for electrical and thermal applications. These efforts were also the result of the need for more effective and compact designs of electrical drives in the automotive industry.

Since copper has high inherent conductivity, it is a highly reflective material when subjected to electromagnetic (EM) radiation. According to Hagens-Rube [2], the metallic reflectance (R), evaluated experimentally, is directly proportional to conductivity and inversely proportional to the frequency of EM wave as in Equation (1), where f is the frequency, $\epsilon_{0}$ is the vacuum permittivity ($8.85\;\times \;10^{-12}\;F/m$), and σ is the conductivity (S/m):

(1) $$R=1-4\;\sqrt{\frac{\;f\;\pi \;\epsilon_{0}}{\sigma }}$$

Since most of the commercial SLM machines are equipped with a continuous laser and feature a wavelength ranging from 1030 nm to 10.6 μm [3,4], materials with higher electrical conductivity, such as pure copper, have shown significant difficulties during printing. This was attributed to the low optical absorption of copper powder, which was reported to be in the range of 32–34% in the near-infrared region (1080 nm) [5,6].

Self-developed high-power, ultra-short pulses (USPs), and short-wavelength (green) laser machines were also explored as alternative methods to deal with copper AM challenges. High laser power in the range of 500 to 1000 W successfully produced high-density parts (over 97%). For example, a fiber laser of 1 kW built on a TRAFAM research lab machine was used to fabricate parts with 97% density at 800 W, although the EC was not reported [7]. Another example was reported in [8], in which an infrared-high power laser of 1 kW was developed in-house at the Politecnico di Milano. A relative density (RD) of 98% was reached. The maximum RD reported in the literature was 99.4% obtained at 800 W laser power, 400 mm/s scanning speed, and 0.07 or 0.09 mm hatch spacing, corresponding to the volumetric energy density range of 740–1120 J/mm^3^ [6]. While high laser power typically improved part relative density, the risk of damaging the laser lens was increased due to significant back reflection of laser exposure. High laser power of 1800 W was also used for printing copper alloys such as Cu-10Zn [9].

Adding alloying elements improves copper processability and reduces the need for redundant laser power despite the negative impact on conductivity. Other relevant examples in the literature include experimental studies using ultra-short laser pulses (USPs) with two central wavelengths of 515 or 1030 nm performed on copper samples to explore the advantage of the high heating rate provided during the short laser–powder interaction times [10]. The capability of a green laser (515 nm) was also examined in printing Cu and Au parts at a power range of 400–1000 W [11,12].

The research reported in the literature was not always restricted to optimizing the laser characteristics. Some researchers considered powder surface treatment to improve the printability of copper. A thin layer (105 nm) of surface oxide was intentionally developed by heating the powder in the open air at 200 °C [6]. Hence, the optical absorptivity of copper oxides was measured at double the optical absorptivity of pure copper. However, the nanometric copper oxide was presented on as-built samples that contributed to less EC. A fluidized-bed method was developed to reduce spontaneous oxidation during transportation and storage. A thin coating layer of polydimethylsiloxane was precipitated on Cu particles, and high laser energy was sufficient to evaporate this layer away [13]. Tin–nickel coating was implemented on the copper powder using the immersion deposition method. The results showed less porosity on the corresponding samples, indicating that the part density achieved from the tin–nickel coating was higher than those samples made from pre-alloyed powder [14]. In general, the coating process is complex and influenced by several parameters regarding the coating variables, such as temperature, PH, potential difference, etc., which will significantly affect the coating properties. Advanced powder preprocessing may indicate an underlying challenge of having inconsistent and unreliable properties of L-PBF products.

Table 1 summarizes the optimum process parameters reported in the literature using commercial L-PBF machines, where process parameters *P*, *v*, *h*, and *t* are laser power, scanning speed, hatch spacing, and layer thickness (*t*), respectively. *E_v_* is the theoretical volumetric energy density.

The current research aims to highlight the ability of commercial SLM machines to deal with highly reflective material. Based on the literature mentioned above, most studies set one or multiple process parameters as constant. However, these parameters may have had a significant effect and are required to be assessed. Accordingly, in this study, a comprehensive range of process parameters was included in the optimization process, in which maximizing RD and EC was the main objective. The most significant process parameters on the part quality were then evaluated. In addition, the surface roughness of the top solidified surface and side surfaces was evaluated in terms of mean arithmetic deviation (Ra). Elemental composition tests were performed on the as-built samples to assess the impact of impurities on the L-PBF part characteristics and their influences on the resulting microstructure. The microstructure evolution along the building direction was investigated. This paper provides a comprehensive experimental study on the process–structure–property relationships during manufacturing pure copper parts by L-PBF using laser power less than 400 W. The resulting part properties, such as electrical conductivity, part chemical composition, and surface roughness, were also evaluated

## 2. Experimental Procedure

### 2.1. Feedstock Material

**Figure 1:**
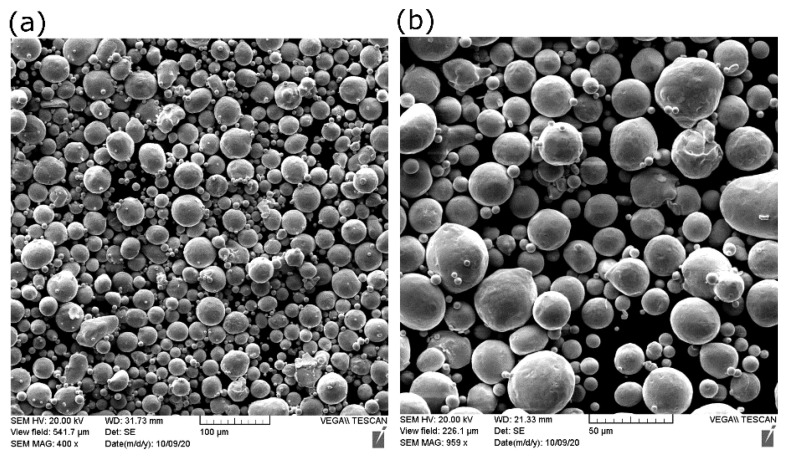
SEM of copper powder used in the current study at (**a**) low and (**b**) high magnification.

A pure copper powder (99.9 wt %), atomized with nitrogen gas, was used as the feedstock material. Scanning electron microscopy (SEM) showed that most Cu particles of the powder were spherical, as shown in Figure 1.

Some small particles called satellite particles were attached to larger ones, along with the existing particle irregularities, causing the flowability to be adversely affected. The chemical composition of the as-received powder was measured using inductively coupled plasma (ICP). The purity of copper was 99.7%. Table 2 shows the characterization results of Cu powder according to the ASTM standards. D10, D50, and D90 are the cumulative distribution of the particle diameters at 10%, 50%, and 90%, respectively.

### 2.2. L-PBF Process

All samples were fabricated using an EOSINT M280 SLM machine, equipped with a 400 W Ytterbium fiber laser. The maximum operating power was 370 W. The nominal laser beam diameter was 100 μm with a continuous wavelength of 1080 ± 20 nm. The building chamber was insulated by a stream of nitrogen gas with a built-in oxygen sensor that ensured an oxygen percentage of less than 0.13% during the SLM process. The building plate was preheated to 200 °C. A high preheating temperature is assumed to be beneficial to cut off a partition of the heat energy required by a laser to reach the melting point. Copper samples were printed directly on top of ground building plates made of steel (DIN 1.2083) since steel has good bonding with copper and maintains most of the input heat energy, particularly for the first few layers [8,13,15].

In this study, the experimental work was performed in two stages. In the first stage, a full factorial design of experiments was implemented to examine the resulting RD, including the laser power *P*, speed *v*, and hatch spacing *h*. Other factors such as the layer thickness t, scanning strategy, and preheating temperature were maintained constant. The detailed process parameters used in this study are illustrated in Table 3, in which the initial set of parameters used in the first stage is underlined. In the second stage, the layer thickness *t*, exposure type, preheating, and scanning orientation were examined one factor at a time.

Since the thermal map surrounding the SLM part leads to variations in properties [21], the samples were distributed on the substrate to ensure no presence of a high concentrated heat zone. Presintering was conducted on 15 coupons aiming more fusion between scan tracks; the surface temperature of these samples was therefore increased before applying primary exposure. Presintering exposure parameters were set at 100 W laser power, 800 mm/s scanning speed, and 100 μm hatch spacing.

**Figure 2:**
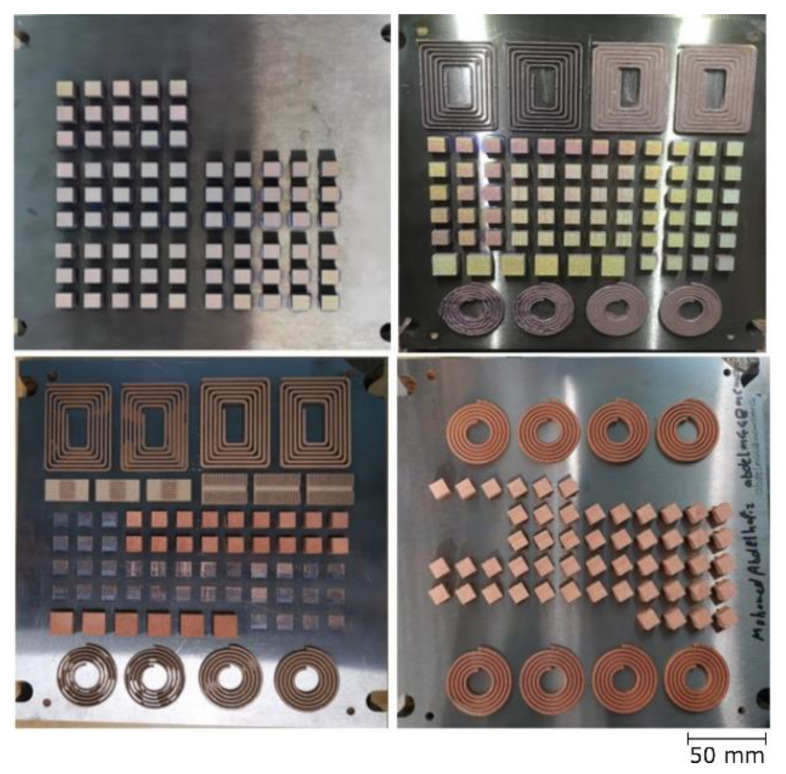
As-built samples arranged before separation from the substrates.

The full coupons used in sample characterization had a side length of 10 mm and a height of 7 mm, with a minimum of 5 mm spacing, as shown in Figure 2. Flat spiral coils, having different shapes (circular and rectangular) and wire diameters of 1 and 2 mm, were printed for EC measurements. The samples were separated via wire EDM.

### 2.3. Sample Characterization

The densification level of the Cu parts was investigated at room temperature using the Archimedes method according to ASTM B962-14. AB204 Mettler balance with a resolution ± 0.1 mg was used to precisely measure the weight of the samples in the air $\left(m_{a}\right)$ and fluid $\left(m_{fl}\right)$. Deionized water was applied as a submerging fluid of density $\rho_{fl}=997.76\;\frac{kg}{m^{3}}$. Using Equation (2), the RD was calculated as sample density relative to Cu bulk density based on the B193-19 standard:

(2) $$\mathrm{RD}=\frac{\rho_{fl}m_{a}-\rho_{a}m_{fl}}{m_{a}-m_{fl}}\frac{1}{\rho_{cu}}\;$$

The as-built samples were examined with a TESCAN VP scanning electron microscope (SEM) using secondary electron at 20 kV (accelerating voltage) and 11 pA (probe current). Surface morphology is a good indication of the wettability and fusion between adjacent scan tracks. The top surface roughness was measured in a transverse scanning direction using a Mitutoyo SJ-410 stylus profilometer, and the average of three measurements was used. For the microstructural analysis, the samples were sectioned parallel to the building direction (Z). Then, the samples were ground using SiC abrasive papers with meshes of 800, 1200, 2400, and 4000, followed by a polishing process using diamond pastes with sizes of 6, 3, and 1 μm.

The elemental composition analysis was performed via energy-dispersive X-ray spectroscopy (EDX) using X-MAX 80 Mm^2^ and INCA software. For comparison, X-ray photoelectron spectroscopy (XPS) was conducted using a PHI QUANTERA II Scanning XPS Microprobe under ultra-high vacuum in the order of 10^−8^ Pa, where the X-ray beam diameter, power, and voltage were 100 μm, 25 W, and 15 kV, respectively. XPS is an excellent tool for elemental analysis and chemical state identification, i.e., oxidation states. However, it is a surface-sensitive technique, so the selected samples were slightly polished once again and stored in a vacuum. In addition to that, the XPS facility had a built-in ion gun used to sputter any possible surface oxidation or other contaminants during storage and handling.

Electrical resistance was measured at room temperature using a Keithley 2400 Source Meter from Tektronix, which had a 0.1 μΩ max resolution and 0.02% error. To eliminate contact resistance, which was more than the resistance of coils themselves, the four-wire Kelvin measurement technique was applied corresponding to the B193-19 standard [22].

## 3. Results and Discussion

### 3.1. Required Volumetric Energy Density

The amount of laser energy required to melt the Cu powder was roughly estimated based on previous studies’ assumptions available in the literature. The volumetric energy density (*E_v_*), which is the amount of energy delivered by the laser beam to the unit volume of the powder, can be expressed as [23]:

(3) $${\;E}_{v}=\frac{P}{v\cdot h\cdot t}$$

**Figure 3:**
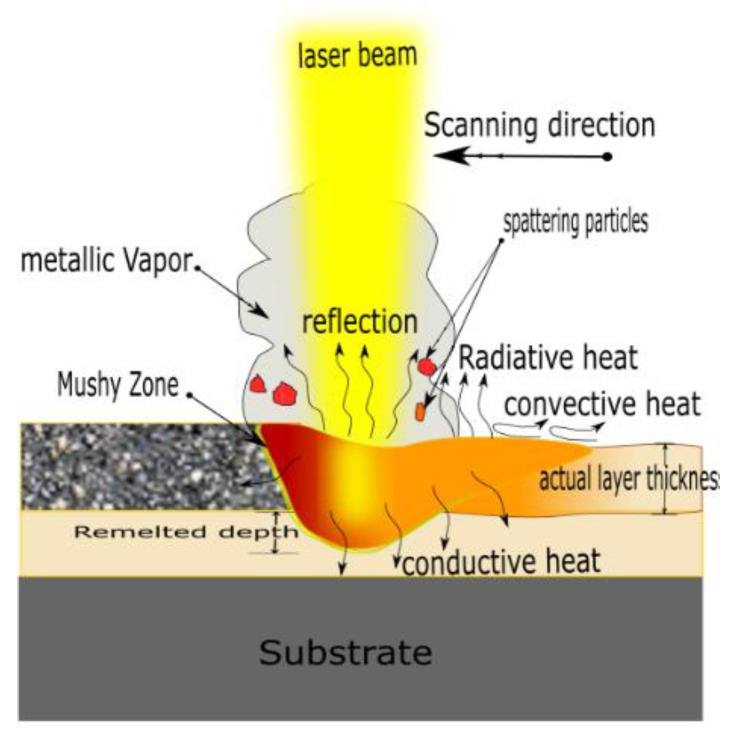
Schematic diagram of heat transfer mechanisms away from the melting pool in L-PBF.

The minimal thermal energy ($E_{t})$ required to melt a unit volume of material is described by Equation (4) [19]:

(4) $$E_{t}=\rho \left(L+C_{p}\left(T_{m}-T_{0}\right)\right)$$

where $\rho ,\;L,\;C_{p},\;T_{m},\;and\;T_{0}$ are copper powder density, latent heat of fusion, specific heat, melting point, and preheating temperature, respectively. Substituting with the thermophysical properties of Cu powder [24] yields $E_{t}$ of 4.8 J/mm^3^, thus *E_v_* should be equal to or greater than this value to ensure that the laser energy can raise the Cu powder temperature to the melting point. However, many factors are not involved in *E_v_*, such as laser distribution, optical absorptivity, heat dissipation, and effective layer thickness, as illustrated in Figure 3. These factors are expressed in Equation (4), resulting in a modified volumetric energy density $\left({E_{v}}_{mod}\right)$:

(5) $$E_{v_{mod}}=\frac{K_{a}\;K_{l}\;K_{d}}{K_{t}}.\frac{P}{v\;h\;t}$$

where $K_{a}$ is the optical absorptivity, which is around 0.44 for Cu powder (particle size less than 100 μm) [25]. $K_{l}$ is the energy loss factor to compensate the amount of heat dissipation and is roughly estimated to be 0.8 [19]. $K_{d}$ is the laser beam distribution factor, which is calculated by integrating the Gaussian distribution function over the laser beam’s radius, and it is equal to 0.865 [19]. $K_{t}$ is the layer thickness factor and is defined by the density ratio of the solidified layer to the powder bed. Due to the reduction of spread powder layer thickness during solidification, the actual powder layer thickness would be greater than the platform displacement. $K_{t}$ is estimated to be 1.753. By equating Equations (4) and (5), the minimum value of $E_{v}$ should be greater than or equal to 28 J/mm^3^. Therefore, all the process parameters combination should meet that requirement to obtained fully melted Cu powder and good quality parts.

### 3.2. Density Measurement

**Figure 4:**
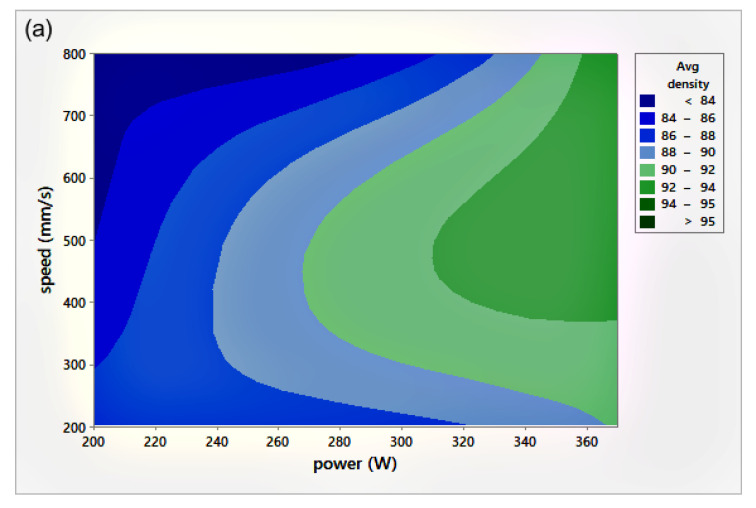

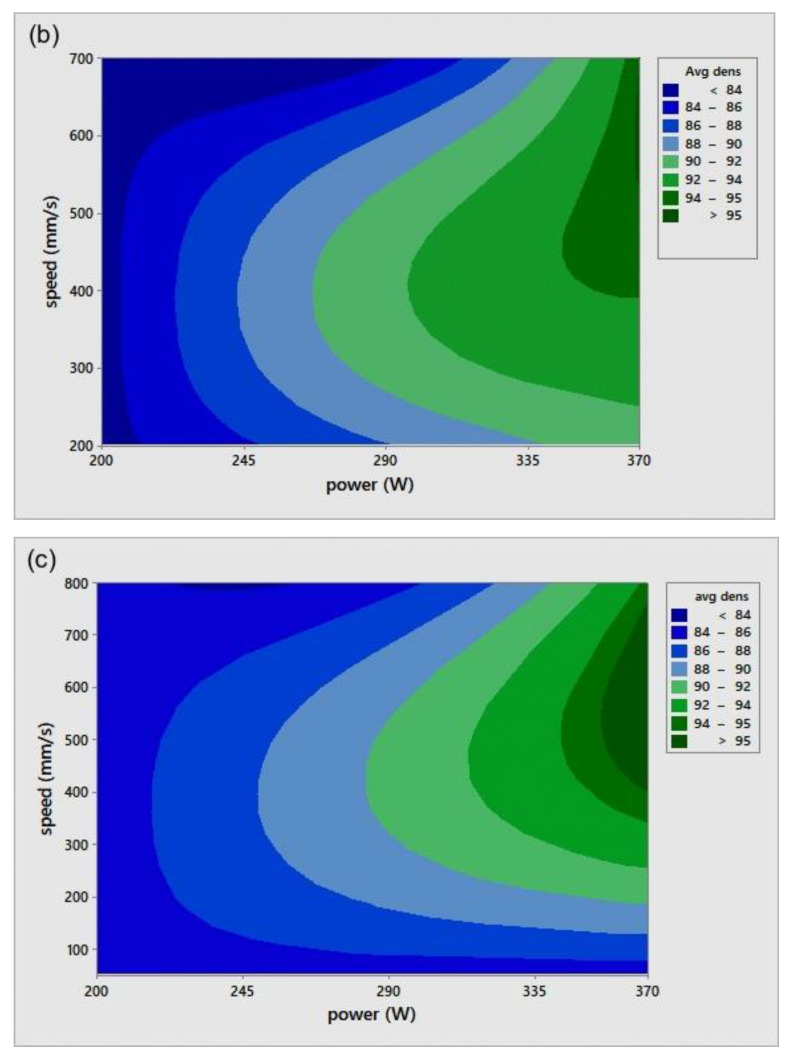
Scan speed (*v*)-laser power (*P*)-relative density (RD) maps for different hatch spacing (*h*) values: (**a**) *h* = 0.05 mm, (**b**) *h* = 0.08 mm, and (**c**) *h* = 0.1 mm.

The relative density (RD) was calculated using the average of three measurements of density for each sample, including the coil-shaped samples. Variation in the scale readings caused an RD error of 0.4%. Figure 4 shows the process parameters (*P-v*) map for different *h*. The *P-v* maps are color-coded, where the greener zone represents the most desirable parameters.

**Figure 5:**
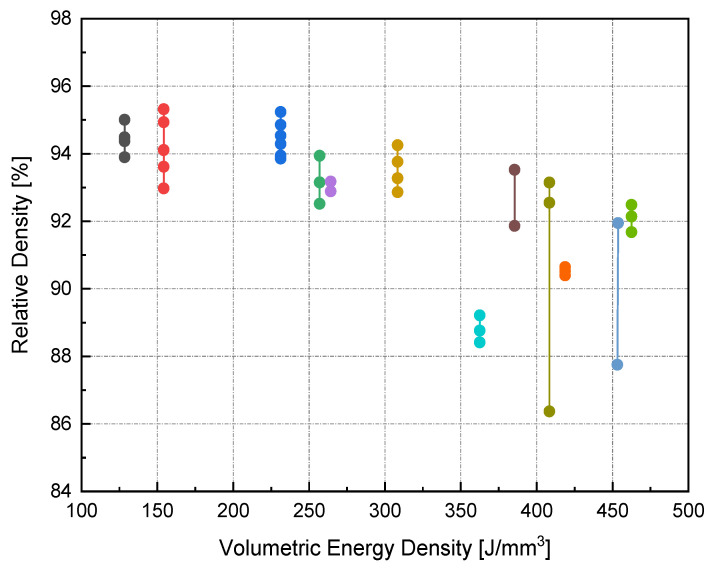
RD variance for groups of identical *E_v_*.

On the other hand, the blue area exhibits the values of *P*, v, and h with lower RD. The range of RD fluctuated from 83–96%. Accordingly, the highest RD obtained was 96% at 370 W laser power, 500 mm/s scan speed, 0.1 mm hatch spacing, and 0.04 mm layer thickness. Neither presintering nor remelting caused RD improvement. Higher RD and good bonding in between the solidified layers were obtained using a scanning orientation of 67°. As expected, and based on previous studies, higher density parts were mainly located at high *P* levels. It was also noted that as the scanning speed increased, the density increased until reaching a value of 500 mm/s; any further increase would result in RD deterioration. In several instances, the combination of process parameters (*P*, *v*, *h*, and *t*) resulted in groups of identical *E_v_*. The associated RD values at the same *E_v_* were observed to be varied (see Figure 5). This was attributed to the different weight of impact for each parameter in the volumetric energy density equation on the part quality.

### 3.3. Effect of Hatch Spacing

**Figure 6:**
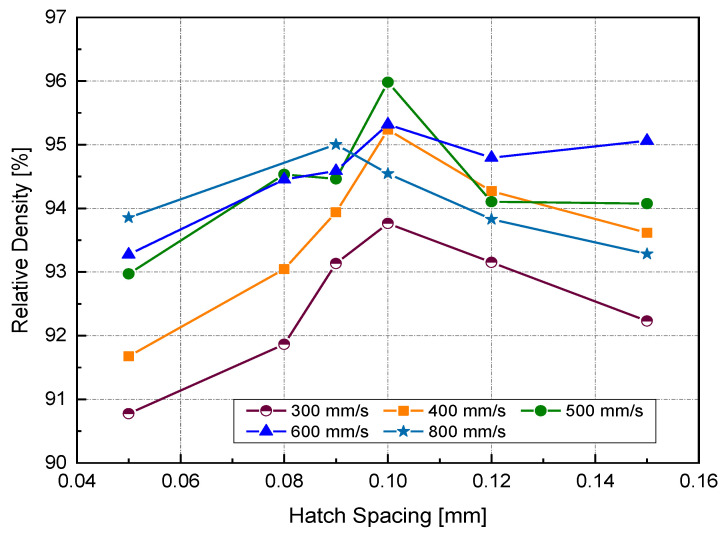
Relative density (RD) at different hatch spacing (*h*) and scanning velocity using a laser power of 370 W.

Figure 6 illustrates the RD variation associated with different *h* and *v* at a laser power of 370 W. In general, up to an *h* value of 0.1 mm, RD increased with increasing *h*. Increasing *h* beyond 0.1 mm resulted in a reduction of the relative density for all scanning speeds, except in the case of high scanning speed, where *v* = 800 mm/s. Referring to the *P-v* maps shown in Figure 4, it was seen that the highest values of RD (darkest green zone) were obtained at *h* = 0.1 mm at a high power level so that the optimum *E_v_* region would be 170–200 J/mm^3^.

It is generally assumed that close hatch spacing provides good fusion between scanned tracks and, thus, better consolidation. Small *h* leads to significant track overlapping. However, the results obtained in this study contradicted that belief. Two reasons might explain this contradiction. The first was the cooling rate associated with track overlapping. Published finite element (FE) modeling work has focused on estimating the resulting cooling rate for various hatch spacing values [26]. A maximum temperature at the center of the melted track (*n*) was obtained in the case of smaller h (75 μm). Because of the high overlapping between two subsequent scanned tracks, a high-temperature gradient will be located across the melt pool, giving rise to a high cooling rate. It was evident in the SEM micrographs of the as-built samples at different h that the depicted cell sizes were inversely proportional to the cooling rate [26]. It was understood that the heat transfer away from the melt pool would be prompted by conduction due to the close adjacent tracks. For high-conductive metals with low h, the heat energy generated in the melt pool will be rapidly dissipated, resulting in insufficient wettability, which may prevent the scanned tracks from being continuous (balling phenomena).

**Figure 7:**
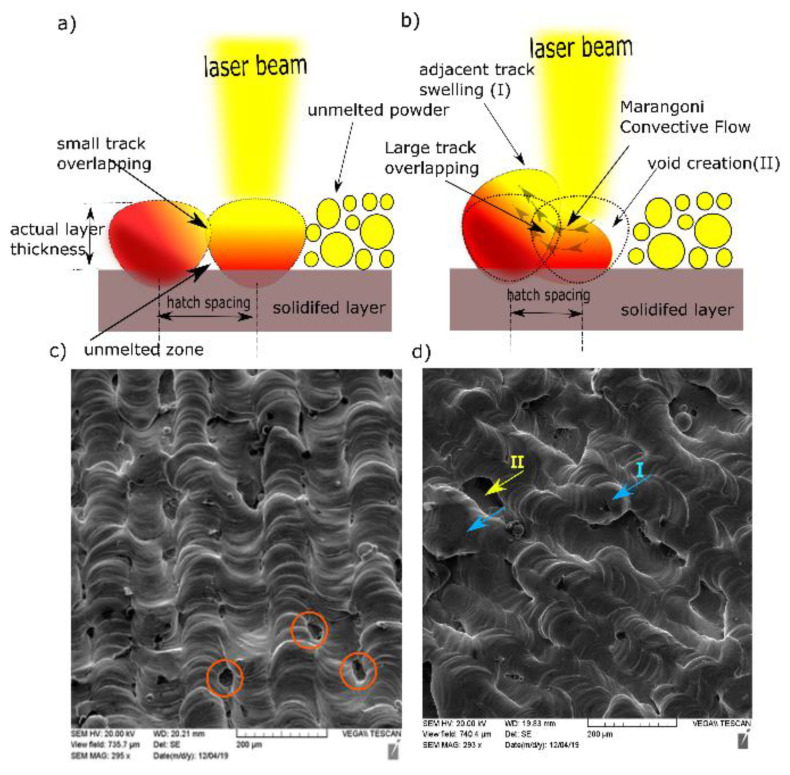
Schematic diagrams for (**a**) small h and (**b**) large h. SEM of top surface at *P* = 370 W, *v* = 350 mm/s for (**c**) *h* = 0.05 mm and (**d**) *h* = 0.1 mm.

Melt pool size specified by the track width is determined by the heat energy input and laser beam diameter at the exposed surface. The power density distribution factor is another important property of the heat source. It is also defined as the distribution factor (f), which describes the density profile of laser power across the beam radius. The commonly used laser sources usually follow axisymmetric Gaussian profiles. As f decreases, the power distribution becomes flatter, and vice versa. Therefore, the intersected partition of the previous track would be heated up and potentially reach the extent of remelting when using a nearly uniform laser along with low h. The flow of liquid metal may occur between the current and preceding tracks due to the spatial variation of surface tension (γ), known as the Marangoni effect [27], as shown in Figure 7b.

The underlying reason behind this variation was the temperature difference between the maximum temperature at the beam axis (less surface tension) and the temperature of the overlapped area (high surface tension). This relationship is quantified by the sensitivity of surface tension to temperature (dγ/dT), which is equal to −0.174 × 10^−3^ N/m.K for Cu [28]. Accordingly, the flow of molten metal was partially directed perpendicular to the scanning direction, which caused swelling (I) in the previous track (*n*−1), as indicated in Figure 7d, as well as void creation (II) between the current solidified track (*n*) and unmelted powder for the track (*n* + 1) [29]. At a laser power of 370 W and a scanning speed of 350 mm/s, the top surface topography showed continuous and stable scan tracks at 0.1 mm hatch spacing (Figure 7c,d). Nonetheless, small gaps were presented between those tracks (circled in red).

### 3.4. Surface Roughness and Dimensional Accuracy

**Figure 8:**
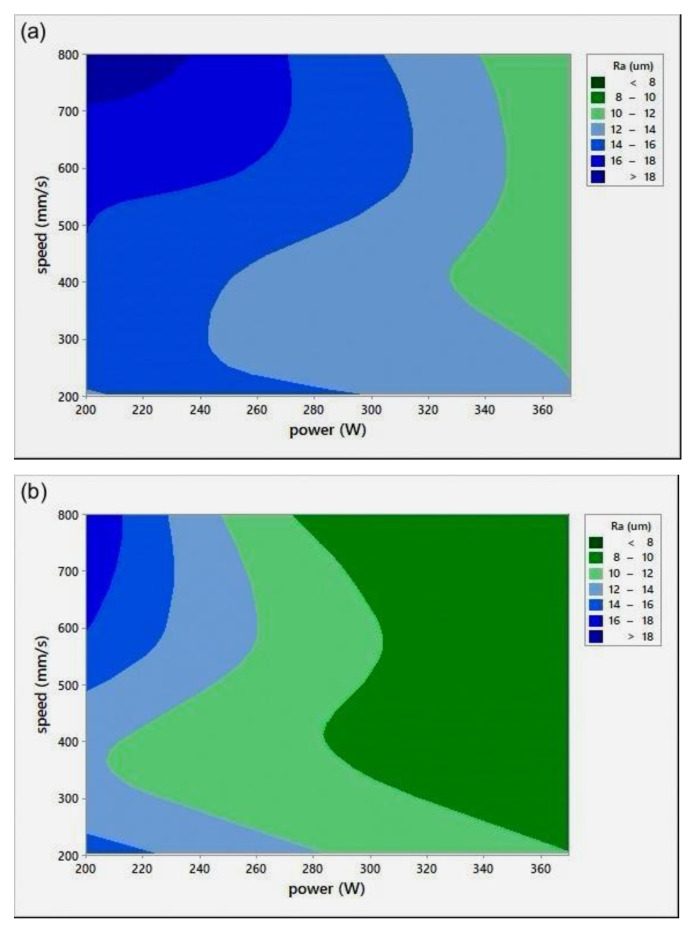

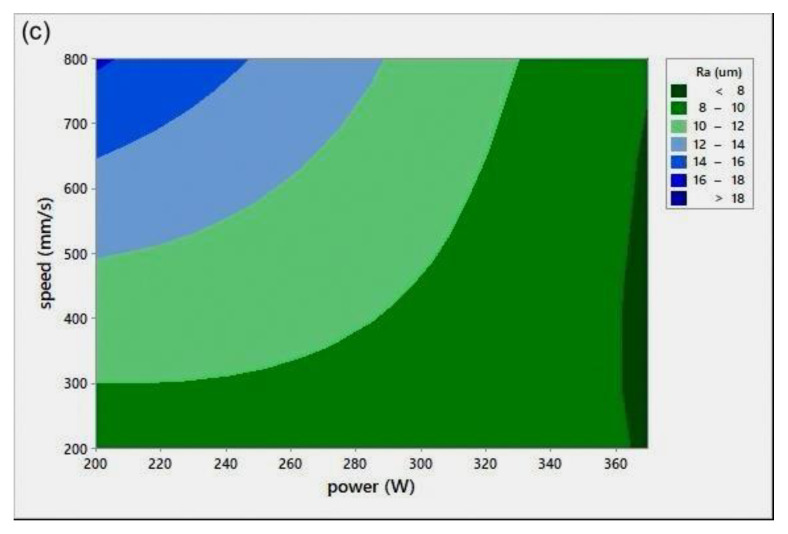
Scan speed (*v*)-laser power (*P*)-surface roughness (Ra) maps for different hatch spacing (*h*) values: (**a**) *h* = 0.05 mm, (**b**) *h* = 0.08 mm, and (**c**) *h* = 0.1 mm.

Surface roughness measurement is typically used to evaluate the quality of the printed surface. Figure 8 shows the *P*-*v* surface roughness maps (Ra) for various *h*.

All Ra measurements were performed in the transverse direction of the scan lines. The figure shows that lower surface roughness values were obtained at high *P* values, a trend similar to the results obtained for relative density (Figure 4). At extremely low RD, it was hard to distinguish scan lines because of their fuzzy shapes. Ra values were found to be quite similar in all directions. It was noted that the low Ra zone existed at high *P*, which was in agreement with the RD results. It was also seen that the target Ra zone (Figure 8c) started at a lower v when compared to the target RD zone in Figure 4c, for *h* = 0.1 mm. The minimum Ra obtained was 8±0.5 μm, equivalent to grade “N8”, a roughness grade number used to categorize the quality of machined surfaces [30].

The dimensional accuracy in the X–Y horizontal plane was defined as the deviation of the sample cross-section area compared to the prespecified dimension. The side length of the as-built sample was measured with a coordinate measuring machine (CMM) with an accuracy of 0.1 μm. With no exception, side length measurements resulted in a positive deviation (greater than 10 mm). The deviation varied between 0.1 mm and 0.22 mm. This resulted in an increase in the corresponding surface area by 3% and 4.5%, respectively. It was noteworthy that the high $E_{v}$ samples possessed a relatively low dimensional accuracy.

**Figure 9:**
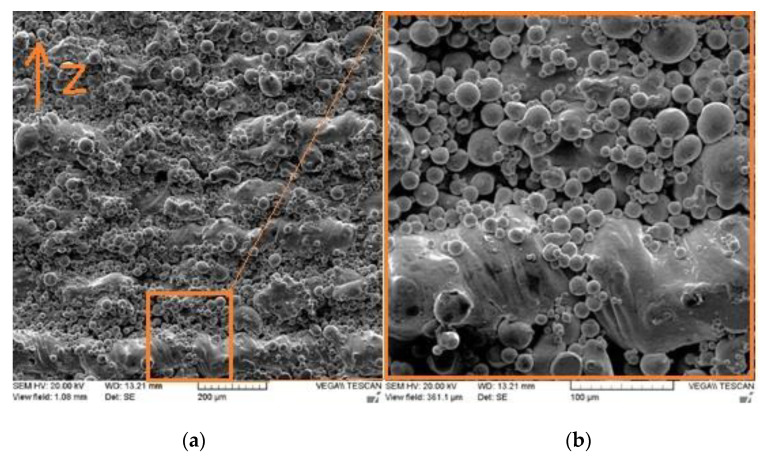
SEM micrographs of the L-PBF Cu part’s side surface at (**a**) low and (**b**) high magnifications.

Figure 9 presents the SEM micrographs of the side surface for the as-built Cu part fabricated using process parameters leading to optimum RD, where the building direction is denoted by z. The figure shows unmelted and/or partially melted particles on the side surface. In contrast, and for the same sample, the top surface quality was much better (Figure 7c). The close contact of the powder, particularly during the liquid phase of layer circumference, gave rise to the affinity of powder particles to unwanted sintering.

**Figure 10:**
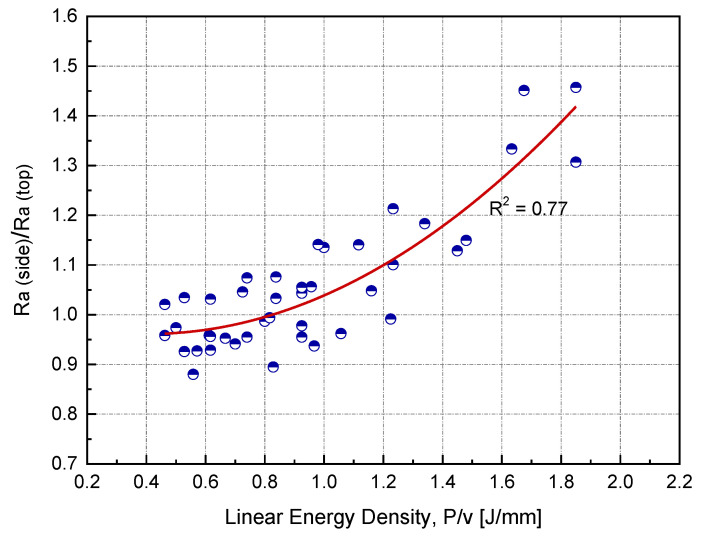
Relationship between the surface roughness ratio and linear input energy at the optimum hatch spacing (*h*) for Cu samples manufactured by L-PBF.

In this study, the sample side surface represented approximately 60% of the total surface area. Side surface roughness is rarely reported in the literature, particularly for copper. Figure 10 shows the ratio of side to top surface roughness as a function of linear input energy (*P*/*v*) for h = 0.1 mm. All side surface roughness measurements were carried out parallel to the building direction. At the optimum linear input energy (0.75 J/mm),$\;Ra_{\;\left(side\right)}$ was found to be higher than $Ra{\;}_{\left(top\right)}$ by 10%. As the input energy increased, the roughness ratio also increased.

### 3.5. Microstructure

**Figure 11:**
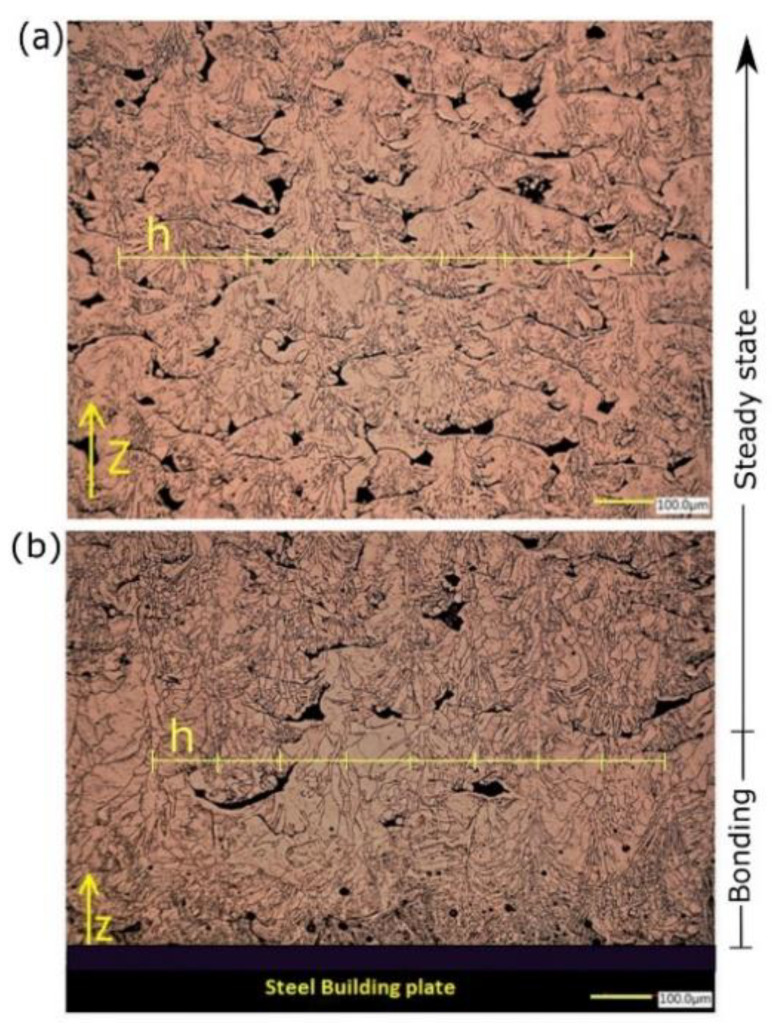
Grain morphology of the L-PBF Cu sample at two locations: (**a**) midpoint and (**b**) close to the steel building plate.

Figure 11 shows the microstructure of copper samples manufactured by L-PBF under process variables of 370 W laser power, 400 mm/s scanning speed, and 100 μm hatch spacing using a KEYENCE digital microscope.

The grain morphology was found to vary depending on the investigated location along the building direction (z). Close to the building plate (bonding zone), the melt pool grain structure (MP) consisted of two distinct zones owning obvious characteristic shapes, starting with columnar-dendritic formed perpendicular to MP boundaries and directed inward. Eventually, the equiaxed grains were formed at the melt pool center, as shown in Figure 11b. On the other hand, at midheight of the sample (steady-state), the in-grain structure examination revealed a formation of mixed cellular and columnar grain growth, which was guided by different solidification conditions (Figure 11a). The columnar grains were periodically repeated and spaced by the same magnitude of *h* = 100 μm, being either in the bonding or steady-state zones. The average size of grain remarkably varied from 10 μm up to 100 μm. In addition, the shape of porosities was irretrievably changed from entrapped vapor (type I), distinguished by an almost spherical shape, to lack of fusion and unmelted powder porosity (type II). Types I and II of porosity were detected in bonding and steady-state zones, respectively. However, type II was observed to be more intense in the steady-state zone. Regarding the melt pool dimension, the melt pool penetration depth was dramatically decreased in the steady-state zone, in which the depth was barely equal to the layer thickness. Nevertheless, melt pool depth could reach the extent of five down-layers, i.e., 200 μm total depth in the bonding zone.

Temperature gradient G, solidification rate R, and the undercooling ∆T are the key factors that govern the solidification mode, microstructure refinement, and consequently, the grain structure development [31]. For a specific material processed by L-PBF, G, R, and ∆T values are determined implicitly by process parameters combination. Even if the process parameters are held constant, grain structure would not be necessarily consistent on both micro and macro scales. For instance, the heterogeneity of the grain morphology observed inside MP (Figure 11b) was attributed to the dynamic G/R ratio [32]. During the rapid solidification of MP, the transition of grain growth from columnar to equiaxed at the MP center was believed to be due to the increased rate of the solid-liquid interface movement, leading to lower G/R [32]. As seen in the bonding zone, the inclination of grain growth direction, perpendicular to MP boundaries, was dominated by maximum heat flux direction, implying that it was aligned with the highest temperature gradient. This phenomenon is well known in polycrystalline material by considering the diffusion of some impurities, such as Fe and C, in the Cu matrix [33,34]. At the bonding zone, a deeper melt pool is believed to be beneficial to promote a good connection between the Cu deposited layer and the substrate; otherwise, it would be rather challenging, particularly for medium laser power machines. However, it may indicate an underlying problem of the gas trapping stimulation due to the evaporation of some diffused alloying elements (impurities) having a lower boiling point.

**Figure 12:**
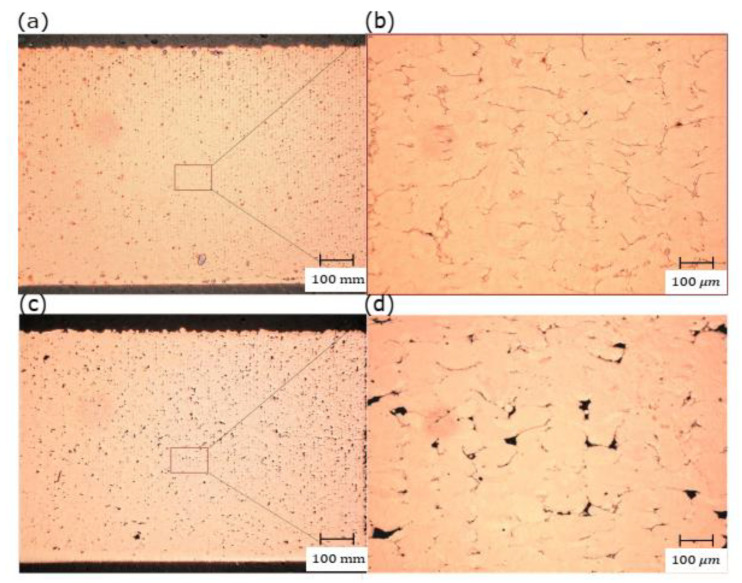
Optical micrographs of the cross-section of L-PBF Cu samples manufactured under 370 W laser power, 600 mm/s scanning speed, and 0.1 mm hatch spacing with two different preheating temperatures: (**a**) 200 °C and (**c**)120 °C; (**b**) and (**d**) are the corresponding high magnifications, respectively.

By increasing the powder bed’s preheating temperature, a significant amount of the heat energy required by the laser beam would be reduced. As a result, the total amount of heat energy gained by preheating and full power of the used laser is supposed to induce sufficient energy in the MP that provides good wettability and fusion. The RD measurements showed a noticeable increase in RD for all the samples fabricated with preheating of 200 °C. Further evincing was observed in the microstructure shown in Figure 12, in which slightly higher porosity was presented with preheating of 120 °C, and thus, the optimum RD was decreased by 3%.

**Figure 13:**
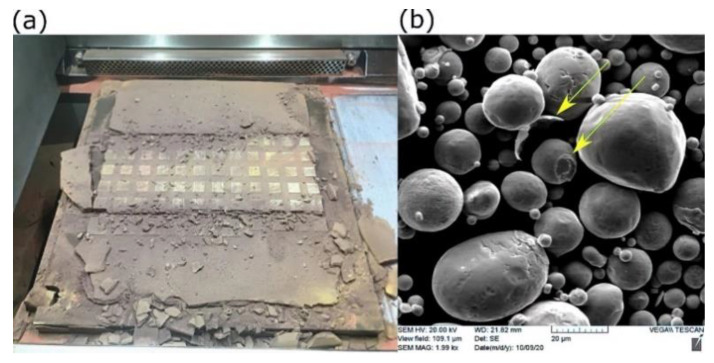
Solid-state sintering of Cu powder at elevated preheating temperature: (**a**) in-field image after printing, and (**b**) SEM of recycled powder (sieved).

However, the challenge of employing a high preheating temperature was increasing the tendency of Cu powder to densify amongst the samples and the interior features that made it quite hard to extract the unmelted powder, as shown in Figure 13.

In this scenario, the solid phase of Cu particles was sintering spontaneously by applying prolonged heating (printing duration). During this stage, neck growth proceeded between neighbor particles to get rid of surface free energy via surface, grain boundaries, and lattice diffusion [35,36].

Although the sintering temperature of Cu is between 700 °C and 1000 °C, the early stage of solid-state sintering could be initiated at low temperatures under compaction. In the current case, the weight of the progressively added powder acted as a compressive force on the powder beneath, which is called in advanced situation “pressure-assisted sintering” [37]. Figure 13b shows SEM of recycled Cu powder after performing mechanical milling and then sieving. Two forms of disintegrated particles are indicated (yellow arrows); a particle with an exfoliated outer shell from its mate and an in-between neck attached to another. Consequently, this may have reduced the flowability of the recycled powder.

### 3.6. Chemical Composition

**Figure 14:**
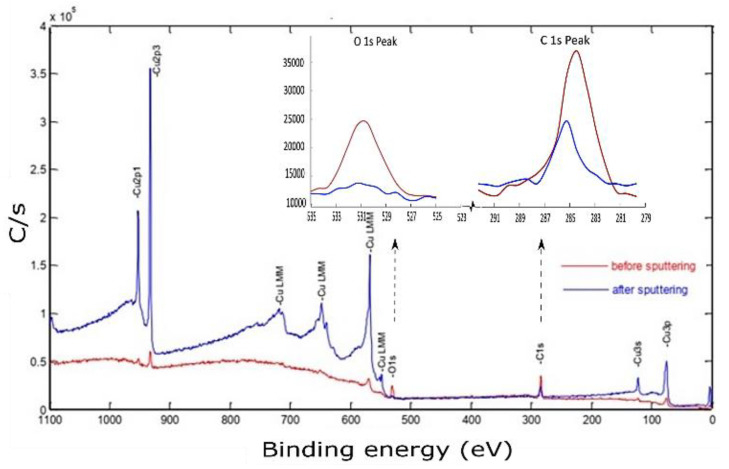
X-ray photoelectron spectroscopy of the Cu sample fabricated by L-PBF under 370 W laser power, 500 mm/s scanning speed, and 0.1 mm hatch spacing, using a polished sample before and after sputtering.

X-ray photoelectron spectroscopy of the Cu sample fabricated by L-PBF under a laser power of 370 W, scanning speed of 500 mm/s, and hatch spacing of 0.1 mm is shown in Figure 14.

The photoelectron emission was attained by hitting the midpoint of the identified sample with an Al-Kα X-ray source $\left(h_{v}=1486.6\;\mathrm{eV}\right)$ at a resolution of 0.8 eV. Although the sample was polished and cleaned before conducting the XPS testing, the spectrum peaks corresponding to the electronic state of the primary element (Cu) were hardly distinguishable. However, other contamination species such as carbon and metal oxides can be noticed at binding energy (BE) of 285 eV and 530.4 eV, respectively. Before sputtering, the high atomic carbon concentration may be ascribed to the presence of hydrocarbon film formation on the sample surface or residual ethanol used for cleaning [38]. After 3 min of 2 keV Ar^+^ sputtering, the characteristic XPS peaks of Cu were revealed. Simultaneously, the C 1s and O 1s peaks had decreased but still existed. The porous nature of the polished sample surface was assumed to provide a good container for contaminations, thus affecting XPS results even after sputtering.

**Figure 15:**
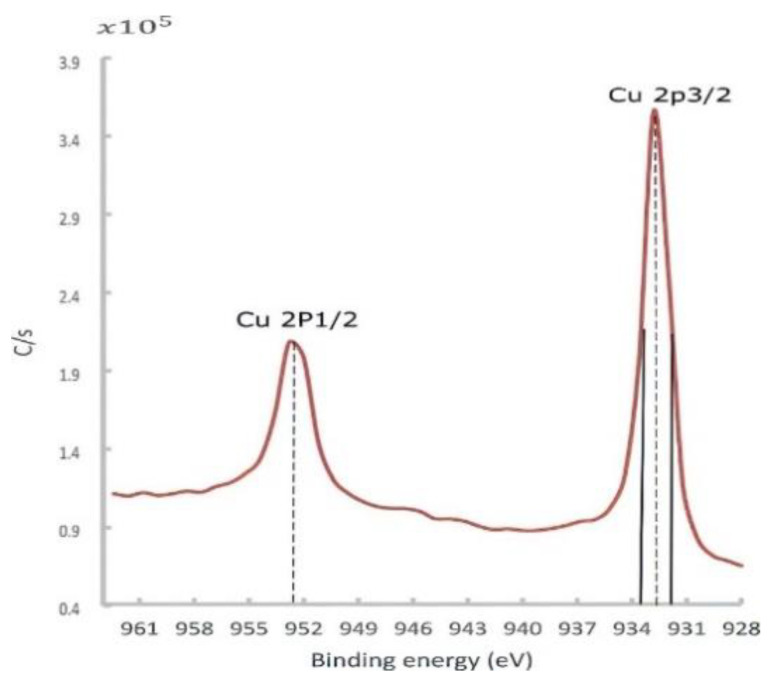
Cu 2px high-resolution spectra for the Cu sample fabricated by L-PBF with a laser power of 370 W, scanning speed of 500 mm/s, and hatch spacing of 0.1 mm, after sputtering.

At low temperatures, a passive layer of the cuprous oxide was rapidly formed, and then the rate dramatically decreased. This well-described phenomenon was formulated by Mott and Cabrera [39,40]. Depending on the temperature, this stable Cu oxide film thickness was varied in the range of 10–100 °A, acting as a barrier that hindered further penetration of oxides. This passive layer also attenuated the photoelectron emission signal coming out from the down metal. The in situ surface-cleaning tool proved its importance in giving an accurate chemical composition. At this point, a high-resolution spectrum of 0.2 eV was utilized to identify the core-level BE and quantify the atomic concentration. The Cu 2p peaks doublet consisted of Cu 2p3/2 and Cu 2p1/2 peaks, as shown in Figure 15.

The Cu 2px peaks along with the O 1s peak could provide valuable information regarding the state of Cu oxides (Cu_2_O and CuO). The Cu 2p3/2 peak, observed at a BE of 932.8 eV with an FWHM of 1.8 eV (Figure 15), was slightly higher than the BE and FWHM of a reference pure copper at the same band, which were found to be 932.3 ± 0.1 eV and 1.5 eV, respectively. In the literature, the Cu_2_O spectrum owned a single peak at 932.4 eV with a narrower FWHM of 1.9 eV.

In contrast, the CuO peak was comparatively wide (3.4 eV), having a BE of 933.2 ±0.1 eV, as well as a satellite peak located between 939 and 946 eV (not shown) [41]. In essence, the obtained spectrum more resembled the Cu_2_O than the CuO spectrum. Furthermore, the O 1s peak emphasized this observation since the O 1s peak was found at a core-level BE of 530.4 eV, which was very close to the O 1s peak of the Cu_2_O phase (529.2 ± 0.1 eV) [41].

**Figure 16:**
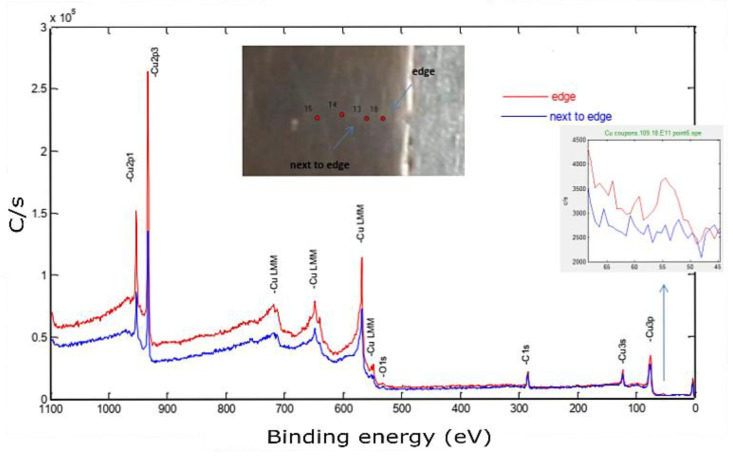
X-ray photoelectron spectroscopy of the Cu sample built by L-PBF (*P* = 370 W, *v* = 500 mm/s, *h* = 0.1 mm). The inset shows a zoom of the Fe3p peak.

Moving to the bonding zone, a certain amount of Fe was introduced, as shown in Figure 16.

**Figure 17:**
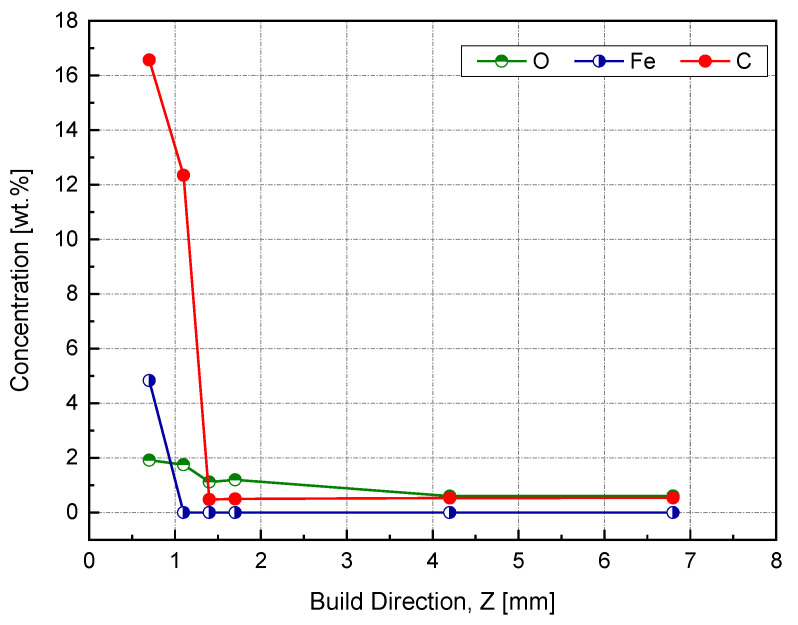
Weight percent of impurities using the XPS peak area along the building direction (Z).

This spectrum was used to identify the impurities in the current sample and specified the quantification regions, which were the doublet peaks of Cu 2p and Fe 3p (because there was a strong overlapping between CuLMM and Fe 2p), C 1s, and O 1s. To track the amount of diffused elements, four equally spaced points were investigated along this zone, as shown in the in situ image of the inspected sample. The next step aimed to determine the atomic concentration of the constitutional elements using the peak areas under high-resolution spectra. The relative sensitivity factor (RSF), stated by Scofield, was utilized to scale the calculated peak area [38]. Figure 17 shows a weight concentration of the impurities along the building direction.

The starting point was at 0.4 mm, which corresponded to 0.7 mm of the actual Z, since the difference between the sample heights before and after wire cutting was 0.3 mm. At this point, we noticed a high Fe content, around 5 wt %, which was above the solubility limit of Fe in Cu, and was attributed to the rapid solidification [33]. The transition from Fe–Cu alloy to Fe-free was supposed to be between 0.7 and 1 mm based on the measurements provided in Figure 16. By entering the steady-state zone, the impurities’ mass concentration significantly dropped while reaching less than 1 wt %, thus producing a high-purity copper sample.

**Figure 18:**
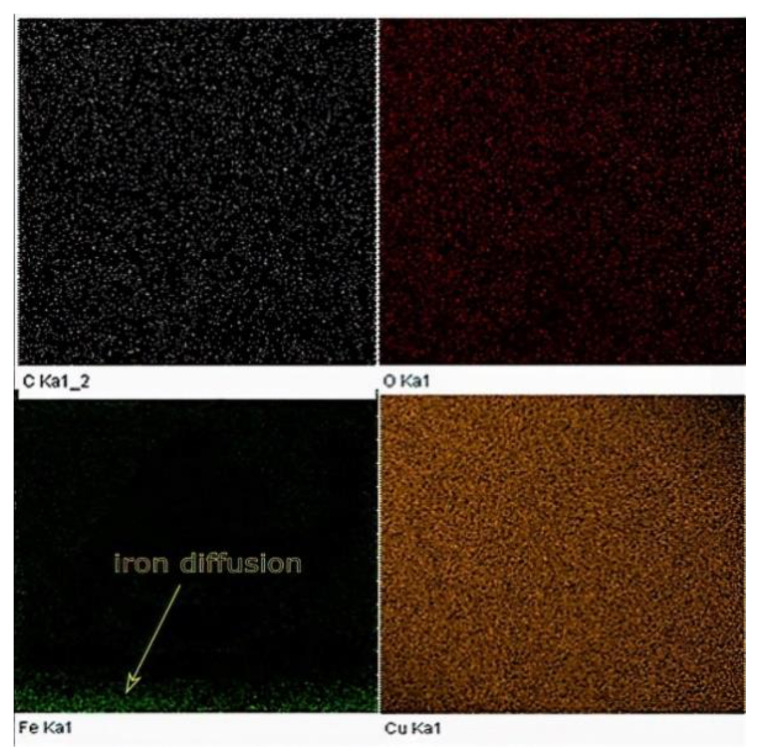
EDX map of the Cu sample built by L-PBF (*P* = 370 W, *v* = 500 mm/s, *h* = 0.1 mm).

For comparison, EDX elemental analysis was performed on the same Cu sample used in the XPS testing. The amount of iron diffusion is presented in the EDX map, as shown in Figure 18.

**Figure 19:**
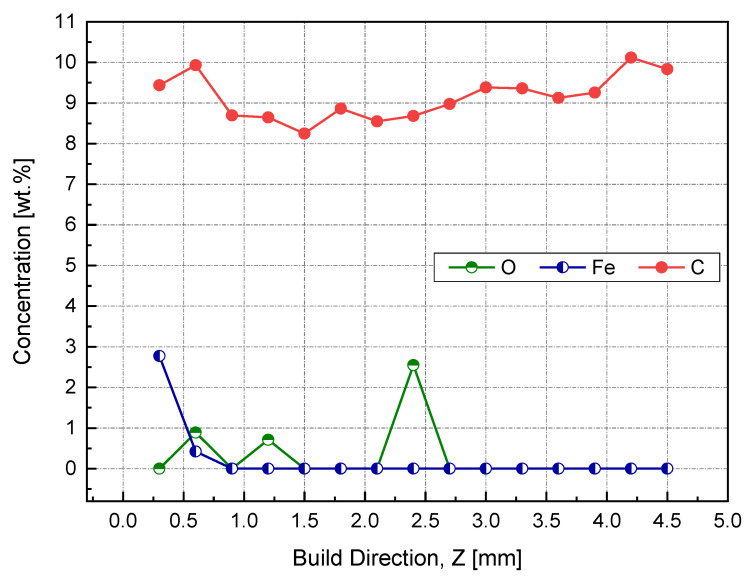
Weight percent of impurities across the height of the Cu sample manufactured by L-PBF, as identified by EDX.

The results obtained from both EDX and XPS were in agreement with the location in which the iron diffusion was stopped. As seen in Figure 19, at a sample height of 0.9 mm, no sign of iron was detected, as indicated in the EDX elemental analysis. Accordingly, printing 1 mm of sacrifice copper substrate would be a good balance between EC and production time, avoiding EC losses due to impurities. It has been speculated that the observed high and almost consistent carbon content across the sample’s investigated height was due to the hydrocarbon contamination. In this regard, the XPS proved its ability to distinguish between carbon from the air contamination and carbon coming from the steel substrate.

### 3.7. Electrical Conductivity

**Figure 20:**
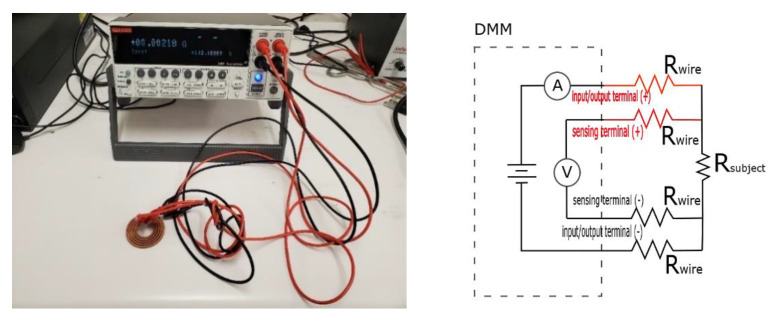
Electrical resistivity measurement of Cu coil made by L-PBF.

The main goal of this study was to maximize the electrical conductivity (EC) of Cu parts made by L-PBF. For that purpose, a set of flat spiral coils were printed using the optimum parameters obtained according to the maximum relative density (RD). The length of all the circular coils was 460 mm, with a 2 mm wire diameter. The resistance of the as-built coils was directly measured using a DC four-wire Kelvin resistance measurement meter, as shown in Figure 20.

EC was calculated using Equation (6) based on the simplified relationship between the current and the voltage drop (V/I), obtaining the coil resistance, namely Ohm’s law [42]:

(6) $$\mathrm{EC}\;\left(S/m\right)=\frac{1}{\rho }=\frac{l}{R\;\;A}$$

where ρ is the resistivity, l is coil length (m), A is the area cross-section (m^2^), and *R* is the resistance (Ω). Another source of *R* error is contact potential; therefore, each sample was measured six times by flipping the terminals every reading. To ensure good contact, surface cleaning was performed by lightly hand-filling the coil terminals. Considering the increase of the cross-section area, the printed coils were measured at different points along the length with a micrometer and had an average diameter of 2.11 mm ± 0.02 mm. The results revealed that the coils had EC values in a range of 3.4 × 10^7^ up to 3.9 × 10^7^ S/m, which corresponded to 60 and 67% IACS, respectively, according to the B193-19 standard [22].

For Cu parts fabricated by powder metallurgy, porosity is one of the prominent contributors to EC deterioration [43], thus optimum RD gained in this study, theoretically, should lead to 95% IACS, according to the density–conductivity relationship [44].

However, in additive manufacturing, metals printed for electrical/thermal purposes also suffer from higher resistivity due to large grain boundaries and lattice dislocation [45]. Microstructural defects such as voids, lack of fusion, and large grain boundaries, as shown in Figure 11, yielded lower EC in the printed coils. Therefore, heat treatment is one of the traditional and helpful postprocessing techniques that has been employed by many researchers for grain growth/coarsening and crystal restoration after the L-PBF process [15]. It was reported that the resistivity of Cu was decreased from 8.18 to 3.69 μΩ.cm by heating at 1000 °C. Additionally, impurities have a negative influence on the EC of copper with no exception, but with disparate impact [24]; for example, 0.05 wt % of phosphorus or titanium would significantly reduce EC by 30% and 40% IACS, respectively. For alloying elements such as silver or zinc, the difference is insignificant; i.e., $∆{\mathrm{EC}}_{\mathrm{Zn}}=-1\;\mathrm{IACS}/0.05\;\mathrm{wt}$. Although the Cu coils, printed directly on top of a steel substrate, had a slightly higher RD than the ones obtained from the full coupon samples (processed at the same conditions), the resulting EC was decreased and not compatible with the RD value, which was caused by the high diffused iron content.

**Figure 21:**
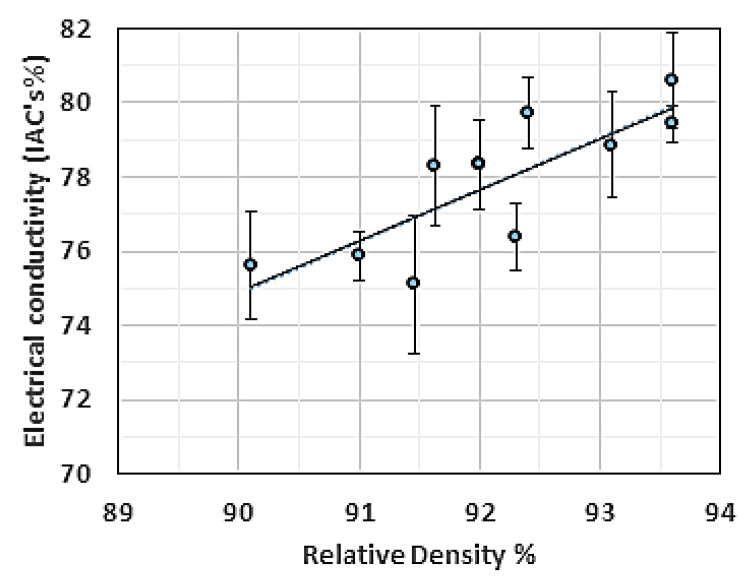
Electrical conductivity (EC) vs. relative density (RD) for the 2 mm Cu coils fabricated by L-PBF.

Another set of coils was reprinted at a 1 mm distance from the substrate using the same length and wire diameter used in the previous experiment. The RD of the reprinted coils demonstrated slightly lower values than the RD obtained from the full coupon samples. However, the corresponding EC measurement indicated a maximum EC of 81% IACS. These results showed that the porosity magnitude was not prevailing in all events; in the current situation, the concentration and species of impurities were more dominant. The EC-RD relationship for the 2 mm Cu coils manufactured by L-PBF is shown in Figure 21.

Even with relatively high purity Cu parts fabricated by L-PBF, the EC outcome was different from the EC value of Cu fabricated by powder metallurgy at the same RD. This may be attributed to the increased amount of grain boundaries. It was also noted that the EC-RD slope was in agreement with the one reported in [43].

As mentioned, the sample size distinctly affected the RD of the reprinted coils compared to the bulky samples. The increased porosity can be attributed to the high surface-to-volume ratio in the case of the Cu coil, leading to a high thermal dissipation and giving rise to a lower peak temperature that can be reached, particularly at the border. Conversely, the sample core held a higher amount of accumulated heat energy and consequently a higher RD [8]. This behavior was emphasized by a further reduction in the wire diameter, where lower RD and EC were acquired from a 1 mm wire diameter coil.

## 4. Conclusions

This work dealt with the process–structure–property relationships of pure copper parts manufactured by laser powder bed fusion. Five levels of laser power of 200, 245, 290, 335, and 370 W; nine scanning speeds of 200, 250, 300, 350, 400, 500, 600, 700, and 800 mm/s; and six hatch spacings of 50, 80, 90, 100, 120, and 150 μm were examined. Layer thickness values of 30 μm and 40 μm, 67° scanning rotation between subsequent layers, and a zigzag scanning strategy were maintained during the processing. A substrate preheating was also considered. Relative density, surface roughness, microstructure, elemental analysis by XPS and EDX, and electrical conductivity were evaluated. The main conclusions are as follows:

1. The highest relative density obtained was 96% when a laser power of 370 W, scanning speed of 600 mm/s, hatch spacing of 100 μm, and layer thickness of 40 μm were employed.
2. Using a maximum permitted laser power of 370 W and different scanning speeds, the relative density was found to increase with hatch spacing until reaching its peak at a hatch spacing of 100 μm, above which further increases in the hatch spacing resulted in relative density deterioration.
3. The minimum surface roughness obtained was 8 ± 0.5 μm, which was comparable to the surface roughness of machined surfaces. At the optimum hatch spacing of 100 μm, the side-to-top surface roughness ratio increased when increasing the input linear energy density.
4. The grain morphology was found to vary depending on the investigated location along the building direction. Close to the building plate (bonding zone), the grain structure inside the melt pool (MP) consisted of two distinct zones owning obvious characteristic shapes, starting with columnar-dendritic formed perpendicular to MP boundaries and directed inwards. The equiaxed grains were formed at the melt pool center. At mid-height of the sample (steady-state zone), a mix of cellular and columnar grains was identified. The average size of grain remarkably varied from 10 μm up to 100 μm.
5. Two types of porosity were detected: spherical (type I) due to entrapped vapor, as well as lack of fusion and unmelted powder porosity (type II). Types I and II of porosity were detected in the bonding and steady-state zones, respectively. However, type II was observed to be more intense in the steady-state zone.
6. The relative density of all samples investigated increased when a preheating of 200 °C was employed during the printing of samples by laser powder bed fusion.
7. During the XPS testing conducted on the polished and cleaned Cu samples, the spectrum peaks corresponding to the electronic state of the constitutional element were hardly distinguishable. However, after sputtering, the characteristic XPS peaks of Cu were revealed.
8. The maximum electrical conductivity (EC) of Cu samples printed by L-PBF was 81% IACS. The impurities in L-PBF were more significant on EC than the porosities presented when using the optimum process parameters.

## Figures and Tables

**Table 1:** Process parameters for printing copper parts made by commercial L-PBF machines and corresponding optimum properties.

Ref.	L-PBF Machine	Process Parameters					Optimum Properties	
		*P* (W)	*v* (mm/s)	*h* (mm)	*t* (mm)	*E_v_* (J/mm^3^)	RD%	EC (IAC’s%)
[13]	Phenix ProX 200	250	800	-	-	-	91	16
[15]	Renishaw PLC AM125	200	300	0.1	0.045	148	86	50.3
[16]	EOSINT M270	195	400	0.08	0.03	203	83	NA
[17]	Sinterstation Pro DM125	200	100	0.12	0.05	333	88.1	NA
[18,19]	EOS M290	200, 300	400, 600	0.08	0.03	208	99	41
[20]	SLM^®^ 125	400	400	0.12	0.03	278	95	98

NA: not available.

**Table 2:** Characterization of the Cu powder used in this study.

Test	Value	ASTM Standard
Sieve analysis (+45 μm)	1.62 wt %	ASTM B214
Laser size diffraction	Dv (10) = 16 μm	ASTM B822
	Dv (50) = 31 μm	
	Dv (90) = 51 μm	
Hall flow	11 s/50 g	ASTM B213
Apparent density	5.07 g/cm^3^	ASTM B212

**Table 3:** List of process parameters.

Process Variables	Levels
Laser power (W)	200, 245, 290, 335, 370
Scanning speed (mm/s)	200–400 (steps of 50), 500–800 (steps of 100)
Hatch spacing (μm)	50, 80, 100, 120, 150
Exposure type	Single, presintering, remelting
Preheating (K)	393, 473
Scanning orientation of each layer	67°, *X*-axis, *Y*-axis
Layer thickness (μm)	30, 40
Scanning pattern	Serpentine (zigzag)
Strip width (mm)	100
Beam offset (mm)	−0.1

Note: Underlined parameters indicate the initial parametes used in the first stage.

## Data Availability

Not applicable.
